# Alzheimer’s Disease and Frontotemporal Dementia: A Robust Classification Method of EEG Signals and a Comparison of Validation Methods

**DOI:** 10.3390/diagnostics11081437

**Published:** 2021-08-09

**Authors:** Andreas Miltiadous, Katerina D. Tzimourta, Nikolaos Giannakeas, Markos G. Tsipouras, Theodora Afrantou, Panagiotis Ioannidis, Alexandros T. Tzallas

**Affiliations:** 1 Department of Informatics and Telecommunications, School of Informatics and Telecommunications, University of Ioannina, Kostakioi, 47 100 Arta, Greece; a.miltiadous@uoi.gr (A.M.); giannakeas@uoi.gr (N.G.); 2 Department of Electrical and Computer Engineering, Faculty of Engineering, University of Western Macedonia, 50 100 Kozani, Greece; ktzimourta@uowm.gr (K.D.T.); mtsipouras@uowm.gr (M.G.T.); 3 2nd Department of Neurology, AHEPA University Hospital, Aristotle University of Thessaloniki, GR54636 Thessaloniki, Greece; afrantou@gmail.com (T.A.); ioannidispanosgr@yahoo.gr (P.I.)

**Keywords:** electroencephalogram, EEG, dementia, Alzheimer’s disease, frontotemporal dementia, classification, k-fold, leave-one-patient-out

## Abstract

Dementia is the clinical syndrome characterized by progressive loss of cognitive and emotional abilities to a degree severe enough to interfere with daily functioning. Alzheimer’s disease (AD) is the most common neurogenerative disorder, making up 50–70% of total dementia cases. Another dementia type is frontotemporal dementia (FTD), which is associated with circumscribed degeneration of the prefrontal and anterior temporal cortex and mainly affects personality and social skills. With the rapid advancement in electroencephalogram (EEG) sensors, the EEG has become a suitable, accurate, and highly sensitive biomarker for the identification of neuronal and cognitive dynamics in most cases of dementia, such as AD and FTD, through EEG signal analysis and processing techniques. In this study, six supervised machine-learning techniques were compared on categorizing processed EEG signals of AD and FTD cases, to provide an insight for future methods on early dementia diagnosis. K-fold cross validation and leave-one-patient-out cross validation were also compared as validation methods to evaluate their performance for this classification problem. The proposed methodology accuracy scores were 78.5% for AD detection with decision trees and 86.3% for FTD detection with random forests.

## 1. Introduction

Dementia is a group of symptoms that can occur when a few groups of brain cells stop working properly and affect desire, ability to react, and movement [1]. It is a neurodegenerative disease that gradually leads to the destruction of nerve cells in the brain. The frequency of occurrence of dementia increases with age, from 1% at ages 60–64 to 24–33% at age 85 and older [2]. Dementia that occurs before the age of 65 is said to be an early-onset dementia, while after 65 it is called dementia with a late onset. Dementia can have many causes and can be reversible or not. The two most common causes of early-onset dementia are Alzheimer’s disease (AD) and frontotemporal dementia (FTD) [3]. AD infects the neurons of the brain and especially the axons by disrupting the neurotransmitters that are responsible for storing memories and transmitting messages to the brain. FTD causes focal degeneration in the frontal, anterior temporal lobe, and islet. FTD’s first description was made by Arnold Pick in 1892 and was composed of these three main symptoms: symptomatic dementia, aphasia, and atrophy of the frontal lobe [4]. Previous attempts to differentiate frontotemporal dementia (FTD) from Alzheimer’s disease (AD) on the basis of reduced concentration and executive dysfunction or reduced memory have often shown uncertain or even contradictory results [5] and much of this controversy may be related to the merging of temporal and frontal cases in FTD, which resulted in blurring of significant neuropsychological discrimination [6]. 

AD and FTD are often misdiagnosed, so the need for a better way of differentiating these diseases is crucial [7]. These two conditions affect different cortical regions. AD mainly affects the hippocampus and posterior temporal and parietal neocortex [8]. FTD affects frontal and anterior temporal regions. Furthermore, the differences of these two conditions regarding the clinical findings are: AD is characterized by amnesia, fluent aphasia, and visuospatial difficulties, while FTD is characterized by changes in personality and behaviour [9].

Neuroimaging methods have contributed significantly to the diagnosis of Alzheimer’s (and FTD) [10].The modern imaging methods used to diagnose dementia are magnetic resonance imaging (MRI) [11], single-photon emission tomography (SPET) [12] which studies the perfusion of the brain with the lipophilic radiopharmaceutical radioactive 99mTc-D,L-hexamethylene-propyleneamine oxime (99mTc-HMPAO) and positron emission tomography (PET) for the study of brain metabolism with fluorinated-deoxy-glucose fluoro-18-fluorodeoxyglucose (18FDG) [13]. In some cases, patients are diagnosed after they have already shown significant neurodegeneration. Thus, accurate prediction of the future onset of Alzheimer’s disease or other dementia types has several important practical applications. It expedites the identification of individuals at high risk of developing dementia to support the clinical development of new therapies, to help plan the overall treatment of the problem [14]. It is therefore important to recognize the disease in its early stages as well as to delay its progression.

Over the last two decades, there has been a significant increase in clinical practice and research interest in electroencephalogram (EEG), as a potential non-invasive tool, sensitive enough for the diagnosis of dementia and classification of its severity. The reason why an EEG-based method for dementia diagnosis and classification is desirable is because of its low cost, its wide availability, and the fact that it is faster than other neuroimaging devices. 

EEG is a technique that records the changes in electrical activity on the cerebral cortex as a function of time. Specifically, it records the change of the electrical postsynaptic potentials produced by brain neurons. EEG signal is acquired by measuring the electrical potentials by electrodes placed on standard, fixed locations on the scalp. EEG spatial analysis is related to the number of electrodes used and their placement or arrangement on the head. The most-used method of placing the electrodes is the international 10–20 system that supports up to 21 electrodes [15].

In recent years, the quantitative EEG (qEEG) has been proven as a reliable clinical tool for the diagnosis and study of brain diseases and cortical disorders such as Huntington’s disease [16], autism spectrum disorders [17], epilepsy [18], and dementia due to Parkinson ‘s disease [19]. In addition, EEG can be used in the differential diagnosis between AD and other diseases leading to dementia, such as vascular brain damage [20,21] and Lewy body dementia [22,23,24,25,26]. Similar methodologies to the one proposed, EEG signal processing and supervised learning classification for recognizing AD patients, have been commonly used in the last decade [27,28,29]. Fiscon et al. used Fourier analysis and wavelet transform to extract EEG features which then were classified by a decision trees algorithm [30]. Safi used the Hjorth parameters along with frequency band decomposition, wavelet analysis, and classification algorithms such as the support vector machine (SVM), K-nearest neighbors (kNN), and regularized linear discriminant analysis (RLDA) to classify AD and healthy subjects [31].

There is a variety of classification algorithms used in the bibliography of EEG classification problems concerning various clinical conditions such as AD [32] or epilepsy [33]. In this work, we used six different classification approaches—meaning lazy classifiers (i.e., kNN), statistical approaches (Naïve Bayes, being the simplest classification), neural networks (i.e., multilayer perceptron), decision trees (C4.5), and one ensemble method (random forests). SVM is a widely used [31,33,34] linear classifier that uses a hyperplane which maximizes the margins (distances from nearest training point) to identify the classes [35]. Artificial neural networks (ANNs) are, along with SVM, the most-used classifiers in BCI research [35]. ANN is a flexible classifier that can be used in a large variety of problems [36]. The most-used ANN for EEG classification is the multilayer perceptron (MLP). In this article, the ANN used was an MLP. kNN is a classification method that assigns a class to an observation according to the dominant class of the k nearest neighbors, calculating its distance to all the observations of the training set. According to Lotte, with enough training samples it can produce nonlinear decisions and approximate any function, but it is not popular for BCI applications because it fails when the experiment has high dimensionality [35]. However, there are multiple experiments in the bibliography that use kNNs [31,37] along with dimensionality reduction [38]. Bayesian classifiers is another category of classifiers used in EEG signal classification [39] for the diagnosis of AD [40], which assign the class with the highest probability, calculated by the Bayes theorem. Finally, decision trees is a classifier that produces a flowchart-like tree structure with each node representing one attribute condition and the attribute selection relies on information gain, while random forests is an ensemble method based on decision trees that, using bagging and boosting techniques, builds multiple decision trees and combines their results to get a more accurate and stable prediction. Both decision trees [30] and random forests [41] implementations are used in the classification of EEG signals for the diagnosis of AD and other dementia types.

In this paper, a methodology for processing and classifying EEG resting-state recordings is proposed. For the selection of the classification algorithm of the methodology the following supervised learning algorithms are compared according to which classifies cases of AD, FTD, and control group instances (CN) with the best accuracy, sensitivity, and specificity: decision trees, random forests, ANNSVM, Naïve Bayes, and kNN.

Accuracy results acquired from both 10-fold cross validation and leave-one-person-out cross-validation methods will also be presented, and the estimated reasons for the differences between their results, as well as the possibility of k-fold cross validation falsely enhancing the accuracy, will be discussed.

## 2. Materials and Methods

The proposed method consists of three stages: data collection, feature extraction, and classification. Initially, EEG signals were acquired from the clinical environment, then they were processed through the OpenViBE software platform (version 3.1.0, Inria Rennes, France) [42] where in statistical and spectral features are extracted. Finally, six classification algorithms were trained and tested on the extracted quantitative EEG features and the evaluation of two learning methods was examined. 

### 2.1. Database Description and Data Acquisition

For this experiment, EEG recordings from 28 participants were obtained from the 2nd Department of Neurology of AHEPA General University Hospital of Thessaloniki. Ten of them were from AD patients, ten from FTD patients, and eight were from healthy age-matched adults that formed the “control” group (CN). The mean ages were 70.5, 67.5, and 68.5 for the AD, FTD, and CN groups, respectively. Table 1 presents the mean age, the Mini Mental State Examination (MMSE) score, which is used to evaluate the cognitive decline and functional performance of patients with AD. [43], the Clinical Dementia Rating (CDR) [44] and the mean duration of disease. For the AD and FTD groups, the mean durations of disease in months were 24 and 26. The EEG device used for the recording of the signals was the Nihon Kohden EEG 2100, using 19 scalp electrodes (Fp1, Fp2, F7, F3, Fz, F4, F8, T3, C3, Cz, C4, T4, T5, P3, Pz, P4, T6, O1, and O2) placed according to the 10–20 international system, while the two reference electrodes (A1 and A2) were placed on the subject’s earlobes (left and right, respectively), for a skin impedance check The impedance value was checked before the recording, ensuring a value below 5 kΩ. All the EEG signals were measured according to the clinical protocol while participants were in sitting position, relaxed, with eyes closed. The recordings were performed with a bipolar anterior–posterior montage (double banana). The filter setting was 0.5–60 Hz. Sampling rate was 500 Hz with 10 μV/mm resolution. Every AD and FTD recording lasted 11–17 min (mean 13) and every control group (CN) recording lasted 20–23 min (mean, 21).

### 2.2. Signal Processing and Feature Extraction

First, the Nihon Kohden EEG 2100 device provided information about any possible artifacts during each EEG recording (blinking, swallowing, any muscle activity). These artifacts were automatically marked and removed. Furthermore, severe artifacts of electrode movement were removed manually by visual inspection. Then, signals were down-sampled from 500 Hz to 250 Hz and a Butterworth band-pass filter (BPF) 0.5–48 Hz was applied to remove power line noise interference at 50 Hz.

Epochs of 5 s with 2.5 s intervals were then extracted. For this study, epochs of 2, 5, and 10 s with 1, 2.5 and 5 s overlap were tested, and the duration of 5 s with 2.5 s overlap was chosen based on accuracy results. Then, time and frequency domain metrics were extracted from each epoch to form the dataset of the classification. Five bandwidth filters corresponding to the five basic EEG rhythms (namely delta, theta, alpha, beta, and gamma) were applied so the energy of each EEG rhythm could be calculated, for the frequency domain metrics, while mean, variance, and IQR were calculated to comprise the time-domain metrics. The frequency bands were defined as: Delta 0.5–4 Hz, Theta 4–8 Hz, Alpha 8–12 Hz, Beta 12–25 Hz, Gamma 25–48 Hz.

**Figure 1:**
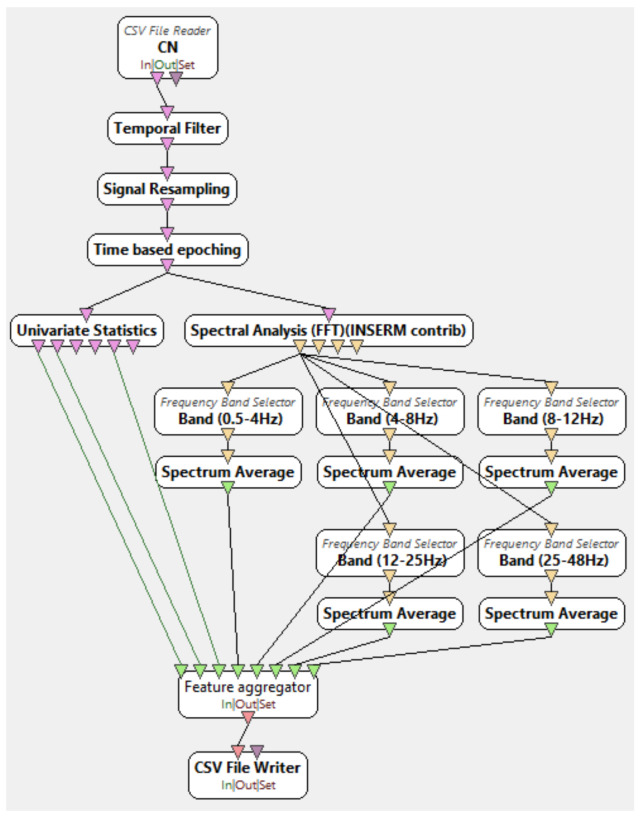
OpenViBE Xml diagram of signal processing and feature extraction.

The use of frequency-domain metrics such as frequency band energy is a widely used method of feature extraction for AD signals classification [30,45] while it is also used on other EEG-based classifications of conditions like epilepsy [46]. On the other hand, time-domain metrics such as mean, variance, and IQR, although not as popular among the EEG classification experiments, have been used before. For example, Tzimourta et al. used mean, variance, and IQR to classify AD and CN signals with accuracy reported up to 91.8% [41]. Therefore, on this basis, mean, variance, IQR, and frequency band energies were chosen as the metrics for AD/FTD/CN classification. Finally, the metrics that were calculated for each electrode created the dataset used in the classification algorithms, which was comprised of 137 headers (8 features * 17 montaged channels + 1 class label). The feature extraction procedure took place at the OpenViBE environment, while the csv manipulation procedures for the accomplishment of compatibility between EDFBrowser (version 1.84, opensource), OpenViBE, and Weka (version 3.8.5, Waikato University, New Zealand) platforms was done using Python scripts. Figure 1 represents the xml diagram of the OpenViBE preprocessing and feature extraction procedure.

### 2.3. Classification

**Figure 2:**
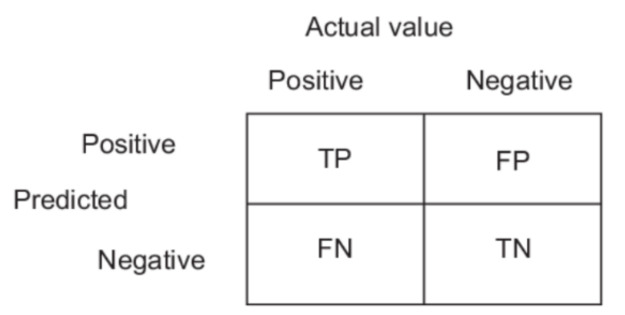
*TP, TN, FP, FN* representation.

The following three classification problems were tested on six classification algorithms, namely decision trees, random forests, ANN, SVM, Naïve Bayes, and kNNs (AD/CN, FTD/CN, and AD/FTD). Also, for each algorithm, the testing method used was leave-one-person-out cross validation and 10-fold-cross-validation, using the implementations on the Weka platform. For each classification case accuracy, sensitivity, and specificity were calculated. Equations (1)–(3) represent the accuracy, sensitivity, and specificity, respectively. *TP*, *TN*, *FP*, and *FN* variables are explained in Figure 2.

(1) $$Accuracy\;=\;\frac{TP\;+\;TN}{TP\;+\;TN\;+\;FP\;+\;FN}$$

(2) $$Sensitivity\;=\frac{TP}{TP\;+\;FN}$$

(3) $$Specificity\;=\frac{TN}{TN\;+\;FP}$$

## 3. Results

Table 2 presents the accuracy, sensitivity, and specificity results of the classification algorithms using the 10-fold cross-validation method. Table 3 presents the accuracy of the leave-one-person-out cross validation. To implement leave-one-person-out cross validation, for each iteration, the whole epoch features of one subject of each class was left out of the training set, which comprised the test set. Then the mean accuracy and the standard deviation (SD) were calculated. Equation (4) represents the SD.

(4) $$\sigma \;=\;\sqrt{\frac{1}{N}\sum_{i-1}^{N}\;{\left(x_{i}\;-\;µ\right)}^{2}}\;\mathrm{with}\;µ\;=\;\frac{1}{N}\sum_{i=1}^{N}\;x_{i}$$

For example, for the AD/CN classification problem, test sets of one AD and one CN subject and training sets of nine AD and nine CN subjects were created accordingly. The sensitivity and specificity of the decision trees (C4.5) and random forests algorithms for the AD/CN and FTD/CN classification problems with leave-one-person-out cross validation are presented at Table 4. 

## 4. Discussion

This study consists of two parts. Firstly, we present a robust method of classifying EEG signals of AD, FTD, and CN participants with decision trees and random forests classification algorithms and validated the results using the most trustworthy leave-one-patient-out cross-validation strategy. Secondly, we evaluate the trustworthiness of the k-fold cross-validation method (which was widely used in previous EEG classification studies) comparing it to the leave-one-patient-out cross-validation method.

Isler, in 2015, observed that using k-fold cross validation in diagnosing congestive heart failure affected the results of the study by falsely enhancing them. The leave-one-patient-out cross-validation method was, as observed, the validation method with the best validity [47]. Hafner, in 2012, proposed that validation methods using samples from the same participant both in the training and in the test set might produce increased accuracy due to bias because the classifier has been trained with samples very similar to the ones that it must be tested, and referred to the leave-one-patient-out cross-validation method as the most realistic one because in prohibits classifier training with patches that belong to the same subject [48].

As far as 10-fold cross-validation strategy is concerned, its accuracy, sensitivity and specificity results for almost all classification problems with almost all classification algorithms were above 90%, far above the accuracy achieved by leave-one-patient out cross validation. This can be explained by the fact that epochs of the same subject were both in the test set and the training set, which allowed the classifier to be trained and tested with same subject epochs so the classification would rely on individual characteristics rather than characteristics that differentiate one class from another class. So, similar to the studies reported previously [47,48] the k-fold cross-validation method is not the optimal method for classifying epoched datasets of EEG signals at this case. However, leave-one-person-out cross-validation method was the most realistic validation strategy because no same-subject epochs were in both the training and in the test set at the same time. Thus, the accuracy of the leave-one-patient-out cross-validation method are considered as the accuracy results of this methodology. 

The best classification algorithm for AD/CN classification was decision trees with accuracy of 78.5% and standard deviation (SD) of 5.8. The random forests was second-best with 77% accuracy and 7.1 SD. Naïve Bayes and kNN had the worst accuracy with 63% and 60%, respectively. It is worth noticing that kNN accuracy with 10-fold was 96%, a fact that can showcase how unfit 10-fold cross validation is for this classification problem (and maybe, also, other epoch-based classification problems).

The best classification algorithm for FTD/CN classification was random forests with 86.3% accuracy and 7.1 SD. Second-best was decision trees with 79.6% accuracy and 11.2 SD. Worst accuracy was again achieved by kNN algorithm at 69.7%.

Finally, the best classification algorithm for AD/FTD classification was decision trees with 73% accuracy and 11 SD. However, all algorithms in this classification problem achieved low accuracies with high SDs. In addition, all algorithms had the tendency to classify most FTD cases as AD, so it may be implied that this method of preprocessing and classification is not well suited for the AD/FTD problem, or that the number of the EEG recordings obtained for this experiment was too small for any of the classification algorithms to be efficiently trained.

Considering decision trees and random forests as the most suitable algorithms for these problems due to the fact that the best accuracies were achieved by them, and leaving aside the AD/FTD problem for which the accuracy of any of the classification algorithms was not satisfactory (low mean with high SD), Table 4 represents the sensitivity and specificity of decision trees and random forests for AD/CN and FTD/CN. With the AD/CN problem, decision trees achieved a sensitivity of 82.4% and specificity of 74%. This means that out of all the epochs of the AD participants of a test set, 82.4% of them were classified correctly as AD while of all the CN epochs, 74% were classified as CN. Respectively, the sensitivity of the random forests algorithm for the FTD/CN problem was 87% and the specificity, 83%.

It should be noted that the number of clinical records for this experiment is considered small, and a bigger subject group could potentially rise the accuracy levels for every one of the three classification problems. The exact settings of decision trees and random forests should also be noted. Decision trees was the J48 implementation with reducedErrorPruning = True, and random forests maxFolds setting was set to 8.

From the study of previous work, a range of studies that analyse the EEG signal in AD and FTD cases can be observed. Researchers have been calculating a variety of statistical characteristics from the EEG recordings, which they used to train their classification models. Table 4 presents some studies that apply statistical analysis techniques and machine-learning algorithms for the study and classification of EEG recordings with FTD and AD cases. In most studies, characteristics are calculated by the software sLORETA [49] and a statistical analysis technique is chosen for the classification of AD or FTD patients and CN. It should be mentioned that in all the studies [37,50], the basic EEG rhythms are further divided and thus the rhythm alpha is found as α1 (8–10 Hz) and α2 (10–12 Hz) [51] and the rhythm beta, which has the largest amplitude, is divided into β1 (12.5–18 Hz), β2 (18.5–21 Hz), and β3 (21.5–30 Hz). The rest of the studies proposed and used one or more types of EEG characteristics to classify AD and FTD. Therefore, the exported EEG characteristics were either frequency-based characteristics, statistical characteristics, or a combination of them. These studies focus on EEG slowing, reduced complexity, reduced synchronization, and neuromodulatory deficit and extract quantitative features to be used as input to several classifiers.

Segmenting EEG signals into time epochs is quite a common technique among the studies, with most of them using 5 s epochs or less. However, a commonly accepted duration size has not been proposed and accepted, with every study choosing a duration in an arbitrarily manner, or by trying multiple duration sizes. The use of overlapping time parts for feature extraction has been shown to improve SNR (signal/noise ratio) characteristics and, consequently, increase classification efficiency [32]. 

Almost every study uses 10-fold-cross-validation as the standard validation strategy, with the most-used classification algorithm being SVM and random forests. The most common classification problems are AD/CN and FTD/CN [37,50] and AD/FTD [52], but AD + FTD/CN has also been studied [34]. 

Table 5 represents a list of previous works on classifying AD, FTD, and CN EEG signals, the methodology they used, and their accuracy, sensitivity, and specificity results.

The results of this study do make the promising insight that the most common forms of dementia could be distinguished based on quantitative EEG characteristics, in an automated way, by machine-learning algorithms. However, further testing of the methodology on a bigger EEG sample of clinical records should be performed to ensure the validity of the method. Moreover, besides AD and FTD, other dementia forms could be studied as to whether they could also be classified by the same methodology. Such forms could include vascular dementia and Lewy body dementia [53], which progresses faster than AD. In addition, the implementation of this methodology on the study and possible distinguishing of the dementia waveforms from the waveforms that characterize seizures, could be of great importance. It is known that Alzheimer’s disease starts by affecting a certain part of the brain and then progresses to lesions throughout the cerebral cortex, so further implementations of the methodology by focusing on specific lobes of the brain [37] should be considered.

At this point, the limitations of this methodology should be addressed. The authors used a relatively small clinical dataset consisting of 18 or 20 subjects for each classification problem. Thus, no clear conclusions can be drawn for the generality of the method. A larger, public dataset could validate the robustness of the proposed method and further demonstrate the generality of the method for EEG signals classification. So, the validation of this experiment with a larger dataset and the extension of this methodology so as the input signals that can be taken from a variety of EEG recorders is of great importance and will be taking place in future work.

Moreover, although the leave-one-out validation method provides a far more accurate estimation of the performance of the classification method than the k-fold cross validation, its computational cost increases as the dataset increases; thus, making it not appropriate for very large datasets. However, keeping in mind that the advantage of the leave-one-out validation is that no common subject epochs are in the training and the test set, when dealing with large datasets this validation method can be manipulated so as the test set does contain more than one subject. By doing so, we keep the distinct advantage of the leave-one-out validation method for epoched datasets, leaving aside the increase in the computational cost.

## 5. Conclusions

In this study, we presented an easy-to-implement method of classifying AD/CN and FTD/CN using the OpenViBE platform for the signal processing and feature extraction and Weka platform for classification, and we compared the classification results of two common validation methods, 10-fold and leave-one-patient-out cross validations. It was observed that the leave-one-person-out cross-validation method was the most trustworthy validation method of the two examined in this study. Decision trees and random forests algorithms achieved the highest accuracies from the six algorithms, for the given method of preprocessing, and for feature extraction. So, random forests is proposed as the classifier for the FTD/CN problem with 86.3% accuracy and decision trees (C4.5) is proposed as the classifier of the AD/CN problem with 78.5% accuracy.

## Figures and Tables

**Table 1:** Database statistical analysis (values in brackets represent standard deviation). The group of patients with Alzheimer’s disease is marked with AD while the group of patients with frontotemporal dementia is marked with FTD. Healthy, age-matched subjects formed the control group (CN).

	Gender (Male/Female)	Age	MMSE	CDR	Disease Duration in Months
AD	6/4	70.5 (7.1)	19.7 (2.76)	1 (0.54)	24 (9.88)
FTD	6/4	67.5 (4.5)	21.5 (1.83)	0.75 (0.26)	26 (9.24)
CN	4/4	68.5 (7.2)	30 (0)	-	-

**Table 2:** Accuracy, sensitivity, and specificity results of six classification algorithms using 10-fold cross validation.

Accuracy of Classification Problem	Decision Trees	Random Forests	AΝΝ	SVM	Naïve Bayes	kNN
AD/CN	96%	99.1%	95%	96.2%	80%	96%
FTD/CN	94.2%	98%	98%	97%	77%	97%
AD/FTD	93.8%	97.7%	90%	91%	69%	95%
**Sensitivity of Classification Problem**	**Decision Trees**	**Random Forests**	**AΝΝ**	**SVM**	**Naïve Bayes**	**kNN**
AD/CN	96.6%	98.6%	96%	98%	94%	96%
FTD/CN	94.1%	98%	98.5%	97%	98%	98%
AD/FTD	95.6%	97.8%	91%	96%	80%	96%
**Specificity of Classification Problem**	**Decision Trees**	**Random Forests**	**AΝΝ**	**SVM**	**Naïve Bayes**	**kNN**
AD/CN	95%	99%	94%	94.4%	58%	96%
FTD/CN	94.4%	98%	95%	97%	62%	99%
AD/FTD	91.3%	97.5%	89.1%	86%	54%	94%

**Table 3:** Accuracy results of six classification algorithms with leave-one-patient-out cross validation.

**Classification**	AD/CN		FTD/CN		AD/FTD	
**Algorithm**	MEAN	SD	MEAN	SD	MEAN	SD
**Decision trees**	78.50%	5.8	79.60%	11.2	73%	11
**Random forests**	77.07%	7.1	86.30%	7.1	64%	12.6
**ANN**	73%	9.4	69.20%	14.1	61%	15.89
**SVM**	68.00%	11.1	75%	12.6	68%	18
**Naïve Bayes**	63%	14	73.80%	25	52%	21.3
**kNN**	60%	11.3	67.30%	9.8	51%	18.2

**Table 4:** Sensitivity and specificity results of decision trees and random forests algorithms.

	AD/CN		FTD/CN	
	Sensitivity	Specificity	Sensitivity	Specificity
**Decision trees**	82.40%	74%	82.20%	77.45%
**Random forests**	78.70%	76%	87%	83%

**Table 5:** Previous work comparison.

Writers	Year	Sample (AD/FTDFTD/CN)	Methodology	Classification Problem	Results		
					ACC	SENS	SPEC
Lindau et al. [50]	2003	16-19-0	Power spectrum of EEG rhythms, cohesion, dominant rhythm	AD/FTD	93.30	-	-
Nishida et al. [37]	2011	19-19-22	EEG rhythms energy, sLORETTA, kNN	FTD/CN AD/CN FTD/AD	85.80 92.80 89.80	55.00 74.00 74.00	84.00 73.00 63.00
Caso et al. [52]	2012	39-39-39	Relative power of EEG rhythms, sLORETTA, ANOVA analysis	AD + FTD/CN AD/FTD	- -	44.87 48.72	85 85
Dottori et al. [34]	2017	13-13-25	Connectivity features, SVM	AD + FTD/CN AD/FTD AD/CN	54.00 73.00 73.00	- - -	- - -
Fiscon D. et al. [30]	2018	86-0-23	Discrete Fourier transform, wavelet analysis, decision trees	AD/CN	83		
Safi M, et al. [31]	2021	51-0-35	Hjorth parameters, discrete wavelet transform, SVM, kNN	AD/CN	97.6		
Proposed Methodology	2021	10-10-8	Energy, mean, variance, IQR, random forests, decision trees	AD/CN FTD/CN	78.5 86.3	82.4 87	74 83

## Data Availability

Not applicable.
